# Correlation of Vascular Calcification With Frailty and Quality of Life in Chronic Kidney Disease Stage 4 and 5 Non-dialysis Patients

**DOI:** 10.7759/cureus.79549

**Published:** 2025-02-24

**Authors:** Tanvi V Thakker, Lalit K Pursnani, Himansu S Mahapatra, Shahina Bano, Muthukumar Balakrishnan, Renju Binoy, Beauty Suman, Mahboob Alam, Abhishek Jha

**Affiliations:** 1 Department of Nephrology, Atal Bihari Vajpayee Institute of Medical Sciences and Dr. Ram Manohar Lohia Hospital, New Delhi, IND; 2 Department of Radiodiagnosis, Atal Bihari Vajpayee Institute of Medical Sciences and Dr. Ram Manohar Lohia Hospital, New Delhi, IND

**Keywords:** chronic kidney disease, frailty, patient reported outcomes, quality of life, vascular calcification

## Abstract

Aim: Vascular calcification (VC) is known to be associated with higher cardiovascular risk in chronic kidney disease (CKD). However, its impact on morbidity factors like frailty and quality of life is understudied. This study aims to address this gap by assessing the correlation of VC with frailty and quality of life.

Methods: Consecutive patients with CKD stage 4 and 5, not on dialysis, were enrolled over one year. Pregnant patients and renal transplant recipients were excluded. VC was assessed using lateral abdominal radiographs, echocardiography, and cardiac computed tomography scans. Frailty was assessed using the Fried frailty phenotype. A Kidney Disease Quality of Life-36 (KDQoL-36^TM^) questionnaire was used for quality-of-life assessment which included physical, mental, and kidney disease-related components. We then investigated the relationships between VC, laboratory parameters, quality of life, and frailty.

Results: A total of 202 patients were enrolled, of which, 26% of patients had detectable VC. Older age, smoking, and coronary artery disease were associated with higher prevalence of VC. Stage of CKD or mineral bone disease markers did not show any significant difference between patients with and without VC. Individuals with VC were significantly more likely to be frail than those without (57.4% vs 30.4%, p-value- 0.001). Patients with VC had significantly lower quality of life scores as compared to those without VC (physical component scores - 45.84±18.98 vs 62.34±22.06; mental component scores - 51.18±20.17 vs 65.99±23.75; kidney disease component scores - 64.90±13.65 vs 74.28±16.32, p-value <0.001).

Conclusion: Patients with VC showed higher prevalence of frailty and significantly impaired quality of life. This highlights the profound clinical implications of VC on functional decline in CKD patients.

## Introduction

Chronic kidney disease (CKD) progression is associated with various abnormalities of bone and mineral metabolism including vascular calcification (VC), collectively known as chronic kidney disease - mineral and bone disorder (CKD-MBD). VC occurs years earlier in CKD patients than in the general population and is associated with increased cardiovascular risk and decreased survival rates [1,2].

Quality of life (QoL) and frailty are considered important measures of disease impact on the lives of CKD patients. These patients experience reduced QoL because of disease complications, treatment burden, and social factors like lower income, lower education, and unemployment [3]. In addition, frailty is highly prevalent even in middle-aged and younger patients with CKD and is linked with adverse clinical outcomes including increased risk of mortality and hospitalization [4,5]. QoL is further worsened in frail CKD patients, probably due to limitations in physical activity and increased disability suggesting that all these factors may be interlinked [6]. However, direct association between VC, frailty, and QoL has not been studied before, particularly in advanced non-dialysis CKD patients. This study aims to address this gap by examining the correlation of VC with frailty and QoL so that it may help us identify appropriate interventions and improve the overall outcomes.

## Materials and methods

Study design

This cross-sectional observational study was conducted over a period of 1st January 2023 to 30th April 2024 at a tertiary care hospital (Dr. Ram Manohar Lohia Hospital, New Delhi) in North India after obtaining approval from the Institutional Ethics Committee.

All consecutive CKD patients with estimated glomerular filtration rate (eGFR) less than 30mL/min/1.73 m^2^ (calculated using the CKD-EPI creatinine equation 2021 [7]) were included. Pregnant patients, patients on any form of renal replacement therapy, and patients with disabilities that precluded physical performance tests necessary for Fried Frailty assessment were excluded. Due to lack of prior research on the specific correlation between VC, Frailty, and QoL in CKD patients, determining a precise sample size for our study proved challenging. Therefore, we screened 260 consecutive non-dialysis CKD patients visiting the nephrology outpatient department, of which 202 patients who fulfilled the inclusion criteria and gave consent for participation were enrolled.

Demographic details and anthropometric measurements (height, weight, body mass index) were recorded. Prior health records were reviewed to identify relevant comorbidities, addictions, and the underlying cause of CKD. Laboratory parameters including hemogram, kidney function tests, serum calcium, phosphate, vitamin D and intact parathyroid hormone levels, serum total protein and albumin levels, urine protein creatinine ratio, etc. were estimated and all the collected data was entered into an Excel spreadsheet for analysis.

Assessment of VC

All the patients underwent screening for VC with a lateral abdominal radiograph and 2D-echocardiography [8]. The severity of abdominal aortic calcification (AAC) on lateral abdominal X-ray was assessed using the Kauppila score which quantifies the AAC observed on a lateral abdominal radiograph from L1 to L4 vertebrae [9]. 2D-Echocardiography was done in standard parasternal and apical views to assess for valvular calcification.

Patients who showed calcification on either lateral abdominal radiograph or echocardiography then underwent further evaluation using an unenhanced cardiac CT scan to look for the presence and extent of coronary artery calcification (CAC). The extent of CAC was quantified with the help of the Agatston score [10]. A score of 0 indicated no identifiable calcification, while scores between 1 and 10 suggested minimal calcification. Scores from 11 to 100, 101 to 400, and over 400 represented mild, moderate, and severe calcification, respectively.

Assessment of frailty and QoL

Frailty assessment was performed using the Fried frailty phenotype [11] which combines five criteria including objective physical assessments and self-reported measures as mentioned below.

Weight Loss

Loss of ≥4.5 kg or ≥5% of total body weight in the last year was considered positive.

Grip Strength

Hand grip strength was assessed in a seated position with the elbow positioned at 90 degrees, supported by the arm of a chair, and the dynamometer supported by the assessor. A hydraulic hand dynamometer was used for grip strength measurement. Hand grip strength measured as a score within the lowest 20% of gender-stratified cut-offs (using the gender-stratified hand grip strength cut-offs proposed by the original frailty phenotype) was considered positive.

Exhaustion

Self-reported score >2 on a scale of 1-4 was considered positive: (i) Exhaustion or fatigue felt very rarely or not at all, (ii) Exhaustion or fatigue felt sometimes, (iii) Exhausted or fatigued most of the time, and (iv) Exhausted or fatigued all the time.

Gait Speed

Time taken to walk 15 feet ≥6 seconds was considered positive.

Physical Activity

A history of the patient's weekly physical activity was recorded, and an estimate of the weekly energy expenditure was calculated. Activity status reported by the patient as a second or third response (amounting to energy expenditure of <383 kcal/week for men and <270 kcal/week for women) was considered positive: (i) I do 30 minutes or more of moderate-intensity physical activities on five or more days per week, (ii) I do 30 minutes or more of moderate-intensity physical activities less than five days a week or during some seasons more than others, and (iii) I am not physically active beyond moving around or walking during activities of daily living.

Patients who were negative for all criteria were considered fit. Patients who were positive for 1 or 2 criteria were considered pre-frail. Patients who were positive for 3 or more of the 5 criteria of the Fried Frailty Phenotype were defined as frail.

QoL was assessed using the KDQOL-36TM questionnaire from RAND Corporation [12]. This questionnaire has been validated for use in the Indian sub-populations in prior studies [3,13]. All the questions were numerically scored according to the standard predetermined scoring system specified by the RAND Corporation. The highest scores (100) were awarded for the best performance and the lowest scores (0) for the poorest performance for each question. The responses were used to calculate scores for 16 different QoL scales including physical functioning, physical role, pain, general health, emotional well-being, emotional role, social functioning, energy or fatigue, symptoms or problem list, effects of kidney disease, burden of kidney disease, work status, cognitive function, quality of social interactions, sleep and social support. These scales were then grouped into three components: physical component score (PCS), mental component score (MCS), and kidney disease-related component score (KDCS).

Statistical analysis

The collected data was analyzed using IBM SPSS Statistics for Windows, Version 25 (Released 2017; IBM Corp., Armonk, New York, United States. The normality of the data was tested by the Kolmogorov-Smirnov test. Quantitative data like age, BMI, hemoglobin levels, eGFR, serum creatinine and electrolytes, vit. D3 and intact parathyroid hormone (iPTH) levels, quality of life scores, etc. were expressed in mean and standard deviation. Differences between groups with and without VC were tested by Student's t-test (unpaired) or the Mann-Whitney 'U' test. Qualitative data like gender, cause of CKD, comorbidities, presence or absence of VC, frailty, etc. were expressed in percentage/proportions. Statistical differences between these proportions in the groups with and without VC were tested by the chi-square test or Fisher's exact test. Spearman correlation was used to assess for correlation of age, BMI, various lab parameters and CKD-MBD markers with QoL components, sub-scales and individual items and to study correlation of various clinical and laboratory parameters with frailty and its components (p-values <0.05 were considered significant, r-values 0-0.39, 0.4-0.79, and 0.8-1 suggested weak, moderate, and strong correlation, respectively). The correlation of the severity of AAC and CAC with QoL scores was also studied using the Spearman correlation test.

## Results

**Figure 1:**
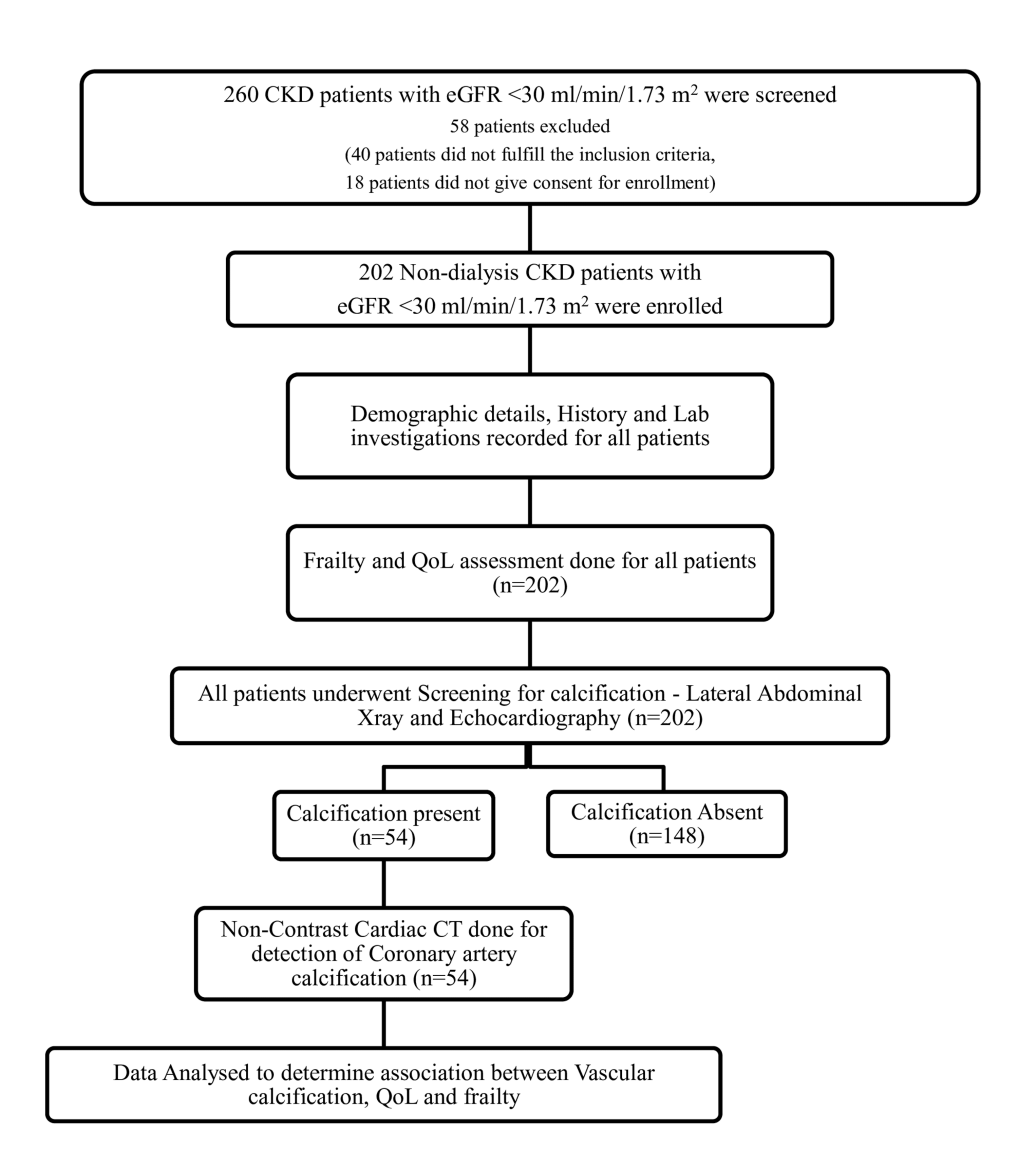
Study algorithm CKD: chronic kidney disease; QoL: quality of life; eGFR: estimated glomerular filtration rate

Out of the 260 non-dialysis CKD patients who underwent screening, 202 patients who fulfilled eligibility criteria and gave consent were enrolled (Figure 1).

The mean age of the study population was 50.77 (±14.95) years, and 56% of patients were male. One-third of the patients were diabetics. The most common causes of CKD in our cohort were diabetic kidney disease (~28%) followed by chronic glomerulonephritis, chronic tubulointerstitial disease, and hypertensive nephropathy. Causes of CKD were unknown in around 20% of patients (Table 1).

**Table 1:** Baseline characteristics of the study subjects (n=202) Descriptive statistics: Quantitative data presented as mean± SD, categorical data presented as number (percentage) BMI: body mass index; CKD: chronic kidney disease; DKD: diabetic kidney disease; CGN: chronic glomerulonephritis; CTID: chronic tubulointerstitial disease; DM: diabetes mellitus; CAD: coronary artery disease; CVD: cerebrovascular disease; eGFR: estimated glomerular filtration rate; UPCR: urine protein creatinine ratio; iPTH: intact parathyroid hormone

Characteristic		Value
Age, years (mean ± SD)		50.77±14.95
Male gender, n (%)		113 (55.9)
BMI (mean ± SD)		23.98±4.35
CKD stage n (%)	IV	105 (52)
	V	97 (48)
Comorbidities n (%)	DM	68 (33.7)
	Hypertension	177 (87.6)
	CAD	34 (16.8)
	CVD	8 (4)
	Obesity	36 (17.8)
	Smoking	43 (21.3)
Serum creatinine, mg/dL		4.03±1.15
eGFR, ml/min/1.73 m^2^		16.95±5.94
UPCR		1.21±1.35
Hemoglobin, g/dL		9.05±1.28
Serum bicarbonate, mEq/L		16.79±3.98
Serum albumin, g/dL		3.34±0.52
Serum calcium, mg/dL		8.49±0.88
Serum phosphate, mg/dL		5.68±3.79
Serum vitamin D3, ng/mL		20.33±10.44
iPTH, pg/mL		505.29±421.49

On screening for VC, 54 (26.7%) patients were found to have calcification. These 54 patients then underwent cardiac CT and 29 of them were found to have CAC (Table 2). The severity of CAC was graded based on the Agatston score measured on cardiac CT; 27.5% of patients had mild calcification, nearly half (44.9%) of patients exhibited moderate calcification and 20.7% showed severe calcification.

**Table 2:** Sites of vascular calcification Descriptive statistics: categorical data presented as number and percentage; *chi-square test used, p-value <0.05 considered significant. The value in bold is considered significant. CKD: chronic kidney disease

Vascular calcification site	Total (n=54)	CKD IV (n=28)	CKD V (n=26)	p-value^*^
Abdominal aortic calcification	31 (57.4)	13 (46.4)	18 (69.2)	0.093
Valvular calcification	27 (50)	16 (57.1)	11 (42.3)	0.272
Coronary artery calcification	29 (53.7)	11 (39.2)	18 (69.2)	0.009

The patients were divided into groups based on the presence or absence of VC and various clinical and lab parameters were compared between the two groups. Age (mean age 59.94 vs. 47.42 years), presence of diabetes (48.1% vs. 28.4%), smoking (33.3% vs. 16.9%), and past history of CAD (35.2% vs. 10.1%) showed a significant difference between patients with and without VC. However, no significant differences were found in CKD-MBD markers between the two groups. The prevalence of VC did not differ significantly among CKD stage IV (51.9%) and V (48.1%) patients (Table 3).

**Table 3:** Difference in clinical and lab parameters between patients with and without vascular calcification *chi-square test used; **Fisher’s exact test used; p-value <0.05 considered significant. The values in bold are considered significant. BMI: body mass index; DM: diabetes mellitus; CAD: coronary artery disease; CVD: cerebrovascular disease; iPTH: intact parathyroid hormone

Parameter studied	Vascular calcification present (n=54)	Vascular calcification absent (n=148)	p-value^*^
Mean age	59.94±12.92	47.42±14.25	<0.001
BMI	24.86±4.28	23.66±4.35	0.12
DM	26(48.1%)	42(28.4%)	0.01
Hypertension	48(88.9%)	129(87.2%)	0.81
CAD	19(35.2%)	15(10.1%)	<0.001
CVD	3(5.6%)	5(3.4%)	0.44**
Obesity	14(25.9%)	22(14.9%)	0.06
Smoking	18(33.3%)	25(16.9%)	0.01
Calcium	8.50±1.10	8.49±0.79	0.90
Phosphate	6.30±7.01	5.45±1.28	0.62
Vitamin D3	21.35±14.54	19.95±8.50	0.99
iPTH	503.55±462.62	505.92±407.13	0.37

Multivariate regression analysis was performed for the variables that showed significant association with univariate analysis. Previous history of CAD and smoking showed a significant positive association with the presence of VC with p-values of 0.003 and 0.046 respectively (Table 4).

**Table 4:** Multivariate analysis for predictors of vascular calcification DM: diabetes mellitus; CAD: coronary artery disease; p-value <0.05 considered significant

Variables	p-value	OR	95% C.I.	
			Lower	Upper
DM	0.085	1.863	0.919	3.779
CAD	0.003	3.441	1.507	7.859
Smoking	0.046	2.181	1.014	4.689

A high prevalence of frailty was found in the study group, with the majority categorized as either prefrail (42.1%) or frail (37.6%). Correlation of various clinical and lab parameters was studied with frailty using the Spearman correlation test. Age (r-value - 0.304, p-value - <0.001) and BMI (r-value - 0.208, p-value - 0.003) showed a moderate positive correlation with frailty score whereas hemoglobin (r-value - 0.249, p-value - <0.001) showed negative correlation. Patients with VC were significantly more likely to be frail than their counterparts without VC (57.4% vs. 30.4%, p<0.001). VC had a major impact on physical activity, grip strength, and gait speed components of frailty.

Patients reported substantially lower scores for physical (57.93 ±22.46), mental (62.03±23.73), and kidney disease-related (71.77±16.17) aspects of QOL. Correlation of various clinical and lab parameters with individual components of the QoL scales was studied using the Spearman correlation coefficient (Tables 5, 6).

**Table 5:** Correlation between study parameters and QoL scales Spearman correlation test used; p-value <0.05 considered significant. The cells that correspond to significant p-values (<0.05) have been highlighted in bold. BMI: body mass index; eGFR: estimated glomerular filtration rate; CKD: chronic kidney disease; PCS: physical component score; MCS: mental component score; KDCS: kidney disease-related component score.

	Age (n=202)		BMI (n=202)		eGFR (n=202)		Hemoglobin (n=202)		Bicarbonate (n=202)	
	r	p	r	p	r	p	r	p	r	p
Physical component scales										
Physical functioning	-0.428	<0.001	-0.166	0.018	0.208	0.003	0.137	0.052	0.062	0.38
Physical role	-0.442	<0.001	-0.076	0.28	0.112	0.112	0.153	0.03	0.107	0.132
Pain	-0.304	<0.001	-0.134	0.057	0.157	0.025	0.083	0.25	0.176	0.012
General health	-0.122	0.08	-0.054	0.444	0.197	0.005	0.203	0.004	0.168	0.017
PCS	-0.418	<0.001	-0.139	0.049	0.189	0.007	0.185	0.009	0.144	0.042
Mental component scales										
Emotional well-being	-0.476	<0.001	-0.095	0.180	-0.008	0.91	0.141	0.046	0.073	0.3
Emotional role	-0.404	<0.001	-0.117	0.098	0.133	0.059	0.163	0.02	0.124	0.08
Social functioning	-0.406	<0.001	-0.066	0.35	0.138	0.051	0.188	0.008	0.142	0.04
Energy/fatigue	-0.401	<0.001	-0.151	0.032	0.168	0.017	0.116	0.102	0.154	0.03
MCS	-0.482	<0.001	-0.111	0.114	0.143	0.042	0.187	0.008	0.148	0.036
Kidney disease component scales										
Symptoms/Problem list	-0.173	0.014	-0.076	0.268	0.243	<0.001	0.182	0.01	0.206	0.003
Effects of kidney disease	-0.546	<0.001	-0.151	0.032	0.043	0.548	0.091	0.2	0.077	0.28
Burden of kidney disease	-0.136	0.054	-0.087	0.221	0.004	0.96	0.089	0.211	0.118	0.095
Work status	-0.443	<0.001	-0.091	0.198	0.135	0.055	0.097	0.17	0.038	0.591
Cognitive function	-0.415	<0.001	-0.089	0.206	0.017	0.82	0.007	0.93	0.08	0.26
Quality of social interaction	-0.406	<0.001	_0.040	0.57	0.07	0.313	0.09	0.21	0.078	0.27
Sleep	-0.380	<0.010	-0.067	0.35	0.06	0.391	0.101	0.153	0.148	0.037
Social support	-0.350	<0.001	-0.076	0.28	0.085	0.231	0.080	0.26	0.132	0.062
KDCS	-0.498	<0.001	-0.114	0.107	0.091	0.2	0.12	0.09	0.126	0.074

**Table 6:** Correlation between CKD-MBD markers and QoL scales Spearman correlation test used; p-value <0.05 considered significant. The cells that correspond to significant p-values (<0.05) have been highlighted in bold. Vit D3: vitamin D3; iPTH: intact parathyroid hormone; PCS: physical component score; MCS: mental component score; KDCS: kidney disease-related component score

	Calcium (n=202)		Phosphate (n=202)		Vit D3 (n=202)		iPTH (n=202)	
	r	p	r	p	r	p	r	p
Physical component scales								
Physical functioning	0.089	0.206	-0.084	0.235	-0.025	0.729	0.056	0.427
Physical role	0.039	0584	-0.029	0.685	-0.064	0.369	0.059	0.402
Pain	0.023	0.745	-0.061	0.392	0.063	0.372	-0.059	0.402
General health	0.073	0.303	-0.112	0.113	0.014	0,841	-0.096	0.175
PCS	0.060	0.393	-0.085	0.229	-0.006	0.934	-0.003	0.972
Mental component scales								
Emotional wellbeing	-0.129	0.068	0.098	0.168	0.079	0.265	0.157	0.026
Emotional role	0.086	0.222	-0.063	0.377	-0.050	0.478	0.030	0.672
Social functioning	0.066	0.353	-0.052	0.459	0.029	0.685	-0.018	0.795
Energy/fatigue	-0.015	0.830	-0.027	0.706	-0.023	0.744	0.001	0.990
MCS	0.044	0.531	-0.042	0.554	-0.024	0.734	0.040	0.575
Kidney disease component scales								
Symptoms/problem list	0.146	0.039	-0.127	0.072	0.112	0.112	-0.104	0.139
Effects of kidney disease	-0.099	0.162	0.011	0.876	-0.091	0.785	0.074	0.294
Burden of kidney disease	-0.019	0.786	-0.002	0.977	0.043	0.544	-0.097	0.170
Work status	-0.019	0.793	0.027	0.704	-0.042	0.554	0.095	0.179
Cognitive function	-0.033	0.640	0.076	0.282	0.077	0.273	0.071	0.314
Quality of social interaction	-0.119	0.092	0.033	0.647	-0.021	0.768	0.039	0.580
Sleep	0.022	0.759	-0.016	0.817	0.064	0.367	0.030	0.676
Social support	-0.031	0.663	0.015	0.829	-0.004	0.953	-0.006	0.927
KDCS	-0.038	0.593	0.029	0.683	0.012	0.862	0.037	0.599

Age was found to have significant negative correlation with all domains of QoL, whereas low hemoglobin and higher BMI were associated with poorer PCS and KDCS.

Although calcium, phosphate, and iPTH levels showed weak correlation with some individual components of physical functioning and kidney disease symptoms, there was no significant impact on broader scales of QoL.

Patients with VC reported significantly worse scores in all sub-scales of PCS, MCS and almost all KDCS components of QOL compared to those without VC (Table 7), indicating a substantial decrement in overall well-being associated with VC.

**Table 7:** Quality of life scores in the study cohort (n=202) Unpaired t-test and Mann-Whitney U test used for comparison between two groups; p-value <0.05 considered significant. The cells that correspond to significant p-values (<0.05) have been highlighted in bold. QoL: quality of life; PCS: physical component score; MCS: mental component score; KDCS: kidney disease-related component score

QoL components	QoL scales	Total study group	Vascular calcification present (n=54)	Vascular calcification absent (n=148)	p-value
Physical	Physical functioning	68.94±2.90	53.80±27.78	74.46±21.33	<0.001
	Physical role	41.46±44.80	19.91±32.72	49.32±46.10	<0.001
	Pain	70.52±22.69	63.66±23.91	73.02±21.78	0.003
	General health	50.79±18.29	46.02±15.64	52.53±18.91	0.014
Total physical component score (PCS)		57.93±22.46	45.84±18.98	62.34±22.06	<0.001
Mental	Emotional wellbeing	73.78±20.03	67.19±18.23	76.19±20.17	0.002
	Emotional role	46.37±45.96	25.93±39.74	53.83±45.93	<0.001
	Social functioning	64.11±25.62	54.40±23.93	67.65±25.38	0.0012
	Energy/fatigue	63.86±19.37	57.22±15.04	66.28±20.23	<0.001
Total mental component score (MSC)		62.03±23.725	51.18±20.17	65.99±23.75	<0.001
Kidney disease	Symptoms/problem list	83.89±11.69	79.55±11.99	85.48±11.21	<0.001
	Effects of kidney disease	61.61±27.65	51.39±26.99	65.35±27.02	0.0013
	Burden of kidney disease	59.40±26.85	51.74±23.87	62.20±27.41	0.016
	Work status	44.05±40.07	30.56±35.58	48.99±40.60	<0.001
	Cognitive function	82.94±15.99	80.86±14.48	83.69±16.48	0.1212
	Quality of social interaction	75.61±18.01	69.01±14.49	78.02±18.61	<0.001
	Sleep	81.30±18.02	77.36±15.84	82.74±18.60	0.025
	Social support	85.36±20.41	78.73±22.43	87.78±19.14	0.003
Total kidney disease component score (KDCS)		71.77±16.16	64.90±13.65	74.28±16.32	<0.001

Correlation analysis was done by using the Spearman correlation coefficient to check for correlation between the severity of CAC (Agatston scoring), AAC (Kauppila score), and various QoL domains. The degree of CAC did not show any statistically significant association with the QoL components (p-value >0.5) whereas AAC showed weak negative correlation with physical, mental, and kidney-specific QoL components (r-values 0.206, 0.179, and 0.203 respectively, p-values <0.05).

## Discussion

VC is an important complication of CKD which is associated with increased cardiovascular risk and mortality. This is a first-of-its-kind study wherein we attempted to assess the relationship of VC with frailty and QoL, which are important measures of impact on CKD on patients’ lives.

The patients in the study cohort were comparatively younger than the Chronic Renal Insufficiency cohort (CRIC) and the German CKD (GCKD) cohort, whereas the mean age was similar to that in the Indian CKD (ICKD) cohort [14-16]. The cause of CKD was unknown in around one-fourth of the patients. Among the rest of the patients, the most common cause was diabetic kidney disease, followed by chronic glomerular disease, hypertensive nephrosclerosis, chronic tubule-interstitial disease, and obstructive nephropathy. These findings were also consistent with those of the CRIC study, ICKD, and GCKD cohorts wherein diabetic kidney disease was the commonest cause of CKD. In the ICKD cohort, the next common cause was tubulo-interstitial disease whereas in the CRIC study, the second most common cause was hypertensive nephrosclerosis [14-16]. Prevalence of hypertension was high in our cohort as evidenced in other major CKD cohorts (>80%). The prevalence of diabetes was similar in the study cohort and the ICKD cohort whereas it was higher in the CRIC cohort [14,15].

The prevalence of VC was notably lower than that reported in previous studies that used computed tomography (CT) for VC detection [17-20]. This discrepancy underscores the superior sensitivity of CT in detecting VC compared to the conventional imaging modalities used in our study. Additionally, cardiac CT revealed CAC in only half of the patients with AAC or valvular calcification, suggesting that the presence of aortic or valvular calcification does not invariably predict CAC. This observation diverges from findings by Bellasi et al., who demonstrated a positive association between the extent of valve and abdominal aorta calcification and the likelihood of various CAC categories in hemodialysis patients [21].

In contrast to the findings of previous studies by He et al. and Garland et al., no significant association was found between VC and serum calcium, phosphate, vitamin D, or iPTH levels [20,22]. This discrepancy could possibly be explained by the smaller sample size of our study and the lower prevalence of calcification in our cohort which might make it difficult to detect subtle associations.

We found a higher prevalence of frailty as compared to previous studies by Shlipak et al. and Roshanavaran et al. [5,23]. However, these studies included fewer individuals with eGFR below 30 ml/min/1.73 m^2^ whereas our cohort exclusively comprised advanced CKD patients with an eGFR<30 ml/min/1.73 m^2^, which likely accounts for the observed higher prevalence of frailty. Unlike previous studies from Brazil and Tukey that reported significant associations between frailty and higher iPTH or lower vitamin D3 levels, MBD parameters did not show any significant correlation with frailty in our cohort [24,25]. Another systematic review by Wu et al. also found no significant association with multivariate analysis [26]. These findings highlight the complex relationship between MBD parameters and frailty. Frailty was significantly higher in patients with VC which was consistent with another study conducted in the elderly population with normal renal function that showed a significant association between aortic arch calcification and frailty [27].

In keeping with the findings of ICKD and CRIC cohorts and previous studies by Manavalan et al. and Mujais et al., patients reported substantially impaired PCS, MCS as well as KDCS scores [3,28-30]. In this study, CKD MBD markers showed no correlation with PCS, MCS, or KDCS whereas another prospective study by Kaur et al. found that phosphate reduction had a significant on PCS suggesting a possible correlation between phosphate levels and QoL [31]. A comparatively narrow range of eGFR and a small sample size could have led to an underestimation of subtle associations in our study. The presence of VC was associated with significantly lower PCS, MCS, and KDCS scores.

In contrast to the clear association between VC and frailty and QoL, our analysis of the subgroup with CAC (n=29) did not reveal significant correlations between CAC severity and QoL scores whereas the subgroup with AAC (n=31) did show a weak negative correlation between severity of AAC and QoL. This discrepancy suggests that while the overall burden of VC significantly impacts QoL and frailty, the severity of CAC may not be a very good predictor of functional decline in these patients.

This is the first study in the literature that studied the impact of vascular calcification on both QoL and frailty in advanced CKD patients who are not yet on dialysis. This population has been underrepresented in previous research in this area. Our findings contribute to the evolving understanding of VC in CKD and its impact on patient-centered outcomes which can contribute to a better understanding of the disease. However, this study has several limitations that warrant consideration. Due to ethical concerns regarding radiation exposure in asymptomatic individuals, we utilized lateral abdominal X-ray and echocardiography for initial VC assessment, with cardiac CT reserved for those with positive findings. This approach likely led to an underestimation of VC prevalence, particularly compared to studies employing CT as the primary screening tool. The limited number of patients with VC reduced the statistical power of our study to examine the impact of VC severity and detect subtle associations with MBD markers. Due to the lack of previous studies in this area, the exact sample size could not be calculated which may have impacted the power of the study. Also, the cross-sectional design prevented the assessment of causal relationships and the evaluation of the impact of VC progression on eGFR decline and long-term outcomes.

## Conclusions

This study highlights the significant impact of VC on both quality of life and frailty. Routine screening for VC should be considered in CKD patients as early identification and implementation of strategies to mitigate progression of VC may help us prevent the functional decline associated with chronic kidney disease. Further research with larger samples and longitudinal designs is needed to unravel the intricate relationships between these factors and to develop targeted interventions aimed at improving the overall well-being and outcomes for people living with CKD.
